# Lorcaserin improves glycemic control via a melanocortin neurocircuit

**DOI:** 10.1016/j.molmet.2017.07.004

**Published:** 2017-07-21

**Authors:** Luke K. Burke, Emmanuel Ogunnowo-Bada, Teodora Georgescu, Claudia Cristiano, Pablo B. Martinez de Morentin, Lourdes Valencia Torres, Giuseppe D'Agostino, Christine Riches, Nicholas Heeley, Yue Ruan, Marcelo Rubinstein, Malcolm J. Low, Martin G. Myers, Justin J. Rochford, Mark L. Evans, Lora K. Heisler

**Affiliations:** 1 Department of Pharmacology, University of Cambridge, Cambridge, UK; 2 Department of Medicine and Wellcome Trust/MRC Institute of Metabolic Science, University of Cambridge, Cambridge, UK; 3 The Rowett Institute, University of Aberdeen, Aberdeen, UK; 4 Instituto de Investigaciones en Ingeniería Genética y Biología Molecular, Consejo Nacional de Investigaciones Científicas y Técnicas, 1428 Buenos Aires, Argentina; 5 Department of Molecular and Integrative Physiology, University of Michigan Medical School, Ann Arbor, MI, USA; 6 Division of Metabolism, Endocrinology, and Diabetes, Department of Internal Medicine, University of Michigan, Ann Arbor, MI, USA

**Keywords:** 5-HT2c receptor, Type 2 diabetes, Hypothalamus, Lorcaserin, Pro-opiomelanocortin (POMC), Melanocortin4 receptor (Mc4r)

## Abstract

**Objective:**

The increasing prevalence of type 2 diabetes (T2D) and associated morbidity and mortality emphasizes the need for a more complete understanding of the mechanisms mediating glucose homeostasis to accelerate the identification of new medications. Recent reports indicate that the obesity medication lorcaserin, a 5-hydroxytryptamine (5-HT, serotonin) 2C receptor (5-HT_2C_R) agonist, improves glycemic control in association with weight loss in obese patients with T2D. Here we evaluate whether lorcaserin has an effect on glycemia without body weight loss and how this effect is achieved.

**Methods:**

Murine models of common and genetic T2D were utilized to probe the direct effect of lorcaserin on glycemic control.

**Results:**

Lorcaserin dose-dependently improves glycemic control in mouse models of T2D in the absence of reductions in food intake or body weight. Examining the mechanism of this effect, we reveal a necessary and sufficient neurochemical mediator of lorcaserin's glucoregulatory effects, brain pro-opiomelanocortin (POMC) peptides. To clarify further lorcaserin's therapeutic brain circuit, we examined the receptor target of POMC peptides. We demonstrate that lorcaserin requires functional melanocortin4 receptors on cholinergic preganglionic neurons (MC4R^ChAT^) to exert its effects on glucose homeostasis. In contrast, MC4R^ChAT^ signaling did not impact lorcaserin's effects on feeding, indicating a divergence in the neurocircuitry underpinning lorcaserin's therapeutic glycemic and anorectic effects. Hyperinsulinemic-euglycemic clamp studies reveal that lorcaserin reduces hepatic glucose production, increases glucose disposal and improves insulin sensitivity.

**Conclusions:**

These data suggest that lorcaserin's action within the brain represents a mechanistically novel treatment for T2D: findings of significance to a prevalent global disease.

## Highlights

• Obesity medication lorcaserin directly improves glycemic control without altering energy balance or body weight.
• Unlike current frontline type 2 diabetes medications, lorcaserin acts within the brain to improve glycemic control.
• Brain Pro-opiomelanocortin (POMC) peptides are a neurochemical mediator of lorcaserin's glucoregulatory effects.
• Lorcaserin increases insulin sensitivity, reduces hepatic glucose production and increases glucose disposal.

## Graphical abstract

**Figure:**
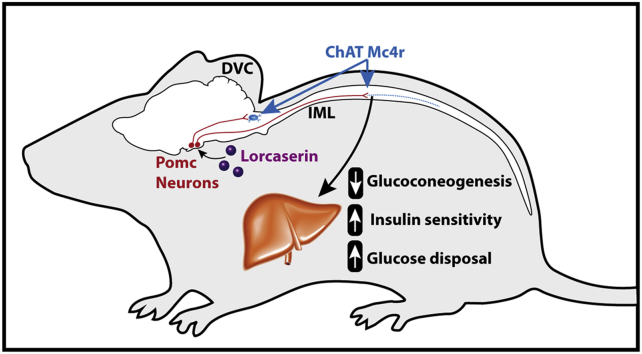

## 1 Introduction

The maintenance of physiologically appropriate levels of glucose is paramount to health and survival. Type 2 diabetes (T2D) is a disease in which insulin fails to provide the normal tight control of blood glucose concentration. The increased global prevalence of T2D underscores the necessity for a more complete understanding of the mechanisms underlying glucose homeostasis to facilitate the identification of new medications. Current frontline T2D medications act at peripheral tissues to potentiate insulin release, insulin action and/or alter glucose absorption and excretion [1]. Here we investigate the therapeutic potential of targeting the brain for T2D treatment given the growing body of evidence implicating brain circuits in glucoregulation [2], [3], [4], [5], [6], [7], [8], [9].

Within the brain, the neurotransmitter and trophic factor 5-hydroxytryptamine (5-HT, serotonin) is required for appropriate energy balance; however, its role in glucoregulation is less clear. Capitalizing on the tight relationship between 5-HT and food intake, medications augmenting the bioavailability of 5-HT were prescribed in the 1990s and 2000s to improve obesity, but were withdrawn from clinical use due to off target effects [10], [11]. Subsequent studies revealed that 5-HT obesity medications primarily elicit therapeutic effects through activation of the 5-HT_2C_ receptor (5-HT_2C_R) subtype influencing the activity of pro-opiomelanocortin (POMC) neurons (POMC^5-HT2CR^) within the brain [12], [13], [14], [15], [16], [17], [18]. Likewise, genetic manipulation of 5-HT_2C_R expression exclusively within POMC neurons influences energy balance, body weight, and glucose homeostasis [5], [18]. The clinical potential of 5-HT_2C_Rs for obesity treatment was realized in the summer of 2013 with the release of the small molecule 5-HT_2C_R agonist lorcaserin (BELVIQ®, Eisai Inc) in the USA. However, given that genetic dysfunction in 5-HT_2C_Rs are associated with T2D in mouse and man alike [4], [19], we considered whether 5-HT_2C_Rs have a direct glucoregulatory function. Recent reports indicate that lorcaserin also improves glycemic control in obese patients with T2D, but this was in association with weight loss [20], [21], [22], [23]. Preclinical compounds with agonist properties at the 5-HT_2C_Rs, such as *m*-chlorophenylpiperazine (mCPP) improve glycemic control without altering energy balance or body weight [24]. Whether lorcaserin also has glycemic effects that may be achieved in the absence of weight loss has not been determined. Here we take advantage of a genetic approach in mice to evaluate whether brain 5-HT_2C_Rs have a direct effect on insulin sensitivity and if so, how this effect is achieved.

## 2 Materials and methods

### 2.1 Mice

*5-HT*_*2C*_*R*^*CRE*^ mice were crossed with ROSA26-stop-enhanced yellow fluorescent protein (YFP) (B6.129X1-*Gt(ROSA)26Sortm1(EYFP)Cos*/J; Jackson Laboratory) mice to create a reporter *5-HT*_*2C*_*R*^*CRE:YFP*^ line as previously described [16]. *Rosa26*^*YFP*^ mice have a *loxP*-flanked STOP sequence followed by a *Yellow fluorescent protein* (*YFP)* gene inserted into the Gt (ROSA)26Sor locus. Intercrossing with *5-HT*_*2C*_*R*^*CRE*^ mice removes the STOP sequence and YFP is visualized in *5-HT*_*2C*_*R*^*CRE*^ expressing cells. *5-HT*_*2C*_*R^CRE^* mice were also crossed with *Pomc*^*NEO*^ mice [3] to generate wild type (WT), *5-HT*_*2C*_*R^CRE^*, ARC *Pomc*^*NULL*^ (*Pomc*^*NEO*^), and restored *Pomc* specifically in 5-HT_2C_R expressing cells (*Pomc*^*5-HT2CR*^) littermates as previously described [16]. *loxTBMc4r* (Jackson Labs) and *ChAT^CRE^* mice were crossed to generate *WT*, *Mc4r^NULL^*, *ChAT^CRE^* and *loxTBMc4r* mice with Mc4rs restored exclusively in cholinergic neurons of the IML and DMV (*Mc4r*^*ChAT*^) as previously described [25]. WT control littermates and C57BL/6J mice (Jackson Labs) were fed 60%-fat diet (HFD; Test Diet, 58Y1) from 3 weeks of age. All other genotypes were fed chow throughout. All mice were maintained on a 12:12 light–dark cycle (lights on at 0700) with *ad libitum* access to chow or HFD diet and water unless otherwise stated. All experiments were in accordance guidelines and approvals of the U.K. Animals (Scientific Procedures) Act 1986.

### 2.2 Drugs

Drugs were prepared in sterile saline and administered intraperitoneally (IP). Lorcaserin (LGM Pharmaceuticals) was administered at 2.5–10 mg/kg.

### 2.3 Glycemic tolerance tests

**Figure 1:**
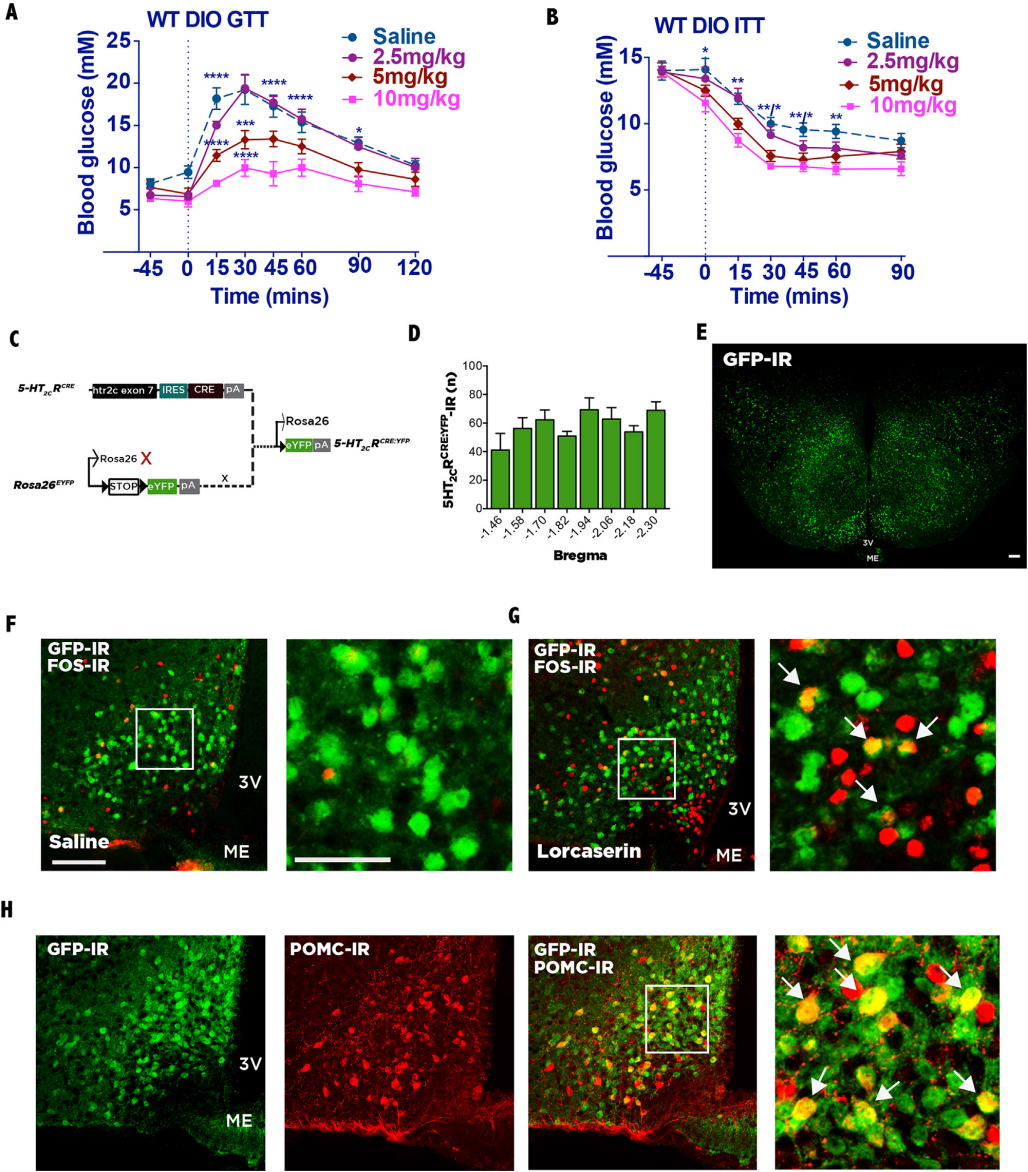
**Lorcaserin improves glucose and insulin tolerance in mouse model of common DIO T2D.** (A) Pretreatment with lorcaserin (5.0 and 10.0 mg/kg, IP) improves glucose tolerance (GTT; glucose 1.0 g/kg, IP) compared to saline in 12 h dark cycle fasted male DIO mice (n = 9). (B) Pretreatment with lorcaserin (5.0 and 10.0 mg/kg, IP) improves insulin tolerance (ITT; 0.75 U/kg, IP) compared to saline in 6 h light cycle fasted male DIO mice (n = 8). (C) *5-HT*_*2C*_*R*^*CRE*^ line intercrossed with a *Rosa26*^*YFP*^ line to visualize *5-HT*_*2C*_*R*^*CRE*^ expressing cells. (D) Bilateral distribution of 5-HT_2C_Rs (GFP-IR) by ARC bregma level (−1.46 to −2.30 mm from bregma) in *5-HT*_*2C*_*R*^*CRE:YFP*^ male and female mice (n = 7). (E) Coronal image of GFP immunoreactivity (IR) in *5-HT*_*2C*_*R*^*CRE:YFP*^ male mouse at the level of the arcuate nucleus of the hypothalamus (ARC). (F–G) Dual-IHC revealed that lorcaserin (7 mg/kg, IP) significantly increases marker for neuronal activity cFOS-IR (red) within ARC 5-HT_2C_Rs (GFP-IR; green; co-expressed yellow) compared with saline treatment in *5-HT*_*2C*_*R*^*CRE:YFP*^ male and female mice (n = 6/treatment). (H) Approximately 40% of POMC-IR neurons (red) co-express 5-HT_2C_R (GFP-IR; green; co-expressed yellow) in *5-HT*_*2C*_*R*^*CRE:YFP*^ male and female mice (n = 6). White box in F, G, and H indicates magnified area; white arrows indicate dual-IHC cells. Scale bar in E, 200 μm. Scale bar in F left panel, 100 μm also applies to G and H; F right panel 50 μm also applies to G and H right panels. *p < 0.05, **p < 0.01, ***p < 0.001, lorcaserin versus saline treatment.

At the onset of the light cycle, food was removed for 6 h to normalize endogenous glucose levels, with the exception of the lorcaserin dose-response glucose tolerance test (GTT; Figure 1A), in which mice were fasted overnight during the dark cycle. Lorcaserin's effects on glucose, insulin, or pyruvate tolerance were assessed via pretreatment with saline or lorcaserin prior to glucose (1 g/kg IP), insulin (0.5 U/kg IP), or pyruvate (1.5 g/kg, IP) bolus, and blood was sampled from tail vein immediately prior to lorcaserin/saline treatment, immediately prior to glucose, insulin, or pyruvate bolus, and 15, 30, 45, 60, and 90 min following bolus administration. Blood glucose was analyzed using an AlphaTRAK glucometer (Abbott Animal Health).

### 2.4 Food intake

Mice were individually housed for 1 week prior to experimentation and during this time received habituation scruffing and IP injection of saline. Mice were treated with saline or lorcaserin (4 or 7.5 mg/kg, IP) 45 min prior to the onset of the dark cycle (lights on/off 0700/1900), and food was removed. At the onset of the dark cycle, food was returned and intake was measured at 1, 3, and 6 h later. Studies were performed using a within-subjects experimental design, with a minimum of a 3 day treatment-free period.

### 2.5 Insulin secretion

To measure insulin secretion, an insulin modified frequently sampled intravenous glucose tolerance test was used. Specifically, DIO male mice were fed 60% fat diet (Test Diet, 58Y1) from weaning to 12 weeks and underwent surgical placement of arterial and venous catheters at 12 weeks as previously described [7], [8]. Four days later, mice were injected with saline or lorcaserin (10 mg/kg, IP) 45 min prior to delivery of glucose (0.75 g/kg over 30 s) via venous catheter. Nine minutes after delivery of glucose, 0.1 U/Kg rapid acting insulin was given. Blood was sampled from freely-moving mice via arterial catheter after glucose bolus and assayed for whole blood glucose (−10, 0, 1, 2, 4, 6, 8, 12, 14, 16, 18, 20, 25, 30, 45, and 60 min) and plasma insulin (0, 1, 2, 4, 6, 10, 12, 16, 20, 30, and 60 min) by RIA (Linco). Blood loss was minimized by intravenous infusion of heparinized blood from donor littermates. Acute phase insulin secretion was calculated as the area under the insulin curve from 0 to 4 min. Insulin sensitivity and glucose effectiveness were also modeled using the minimal model [26].

### 2.6 Hyperinsulinemic-euglycemic clamp

Hyperinsulinemic-euglycemic clamps were conducted as previously described [27]. Animals were anesthetized by a combination of 6.25 mg/kg acetylpromazine, 6.25 mg/kg midazolam, and 0.31 mg/kg fentanyl, IP. An infusion needle was placed into the tail vein and D-[^3^H] glucose (specific activity: 10–20 Ci (370–740 GBq)/mmol) was infused at a rate of 0.006 MBq/min for 60 min to achieve steady-state levels. Thereafter, insulin (Actrapid; Novo Nordisk) was infused at a constant rate of 0.09 mU/min after a bolus dose of 3.3 mU and d-[^3^H]-glucose was continued at a rate of 0.006 MBq/min. A variable infusion of 12.5% d-glucose was used to maintain blood glucose at euglycemic (basal) levels. Blood glucose was measured with an AlphaTRAK glucometer (Abbott Animal Health) every 5–10 min from superficial tail vein and glucose infusion adjusted accordingly. After 50 min from the start of the insulin infusion, [^14^C]-2-Deoxy-glucose-phosphate (Specific Activity: 250–350 mCi (9.25–13.0 GBq)/mmol, IV) was administered to assess tissue-specific glucose uptake. Steady state was reached after 90 min and blood samples were taken at 10 min intervals over 30 min to determine steady-state levels of [^3^H]-glucose. Mice were killed by cervical dislocation and organs were removed and frozen. Hematocrit was measured at baseline and after the clamp (no significant changes were observed between each genotype and their littermate controls, data not shown). To measure plasma [^3^H]-glucose, proteins were precipitated with trichloroacetic acid (final concentration 10%), centrifuged and supernatant dried and re-suspended in water. The samples were counted using scintillation counting (Hidex Scintilation counter, LabLogic). Tissue samples were homogenized (∼5–10% wet wt/vol, depending on tissue) in 0.5% percholic acid, centrifuged, supernatants neutralized, and [^14^C]-2-Deoxy-glucose-phosphate precipitated using BaOH/ZnSO4. The glucose turnover rate (μmol/min) was calculated under steady-state clamp conditions as the rate of tracer infusion (dpm/min) divided by the plasma specific activity of [^3^H] glucose (dpm/μmol). The hyperinsulinemic hepatic glucose production was calculated as the difference between the tracer-derived rate of glucose appearance and the glucose infusion rate.

### 2.7 Immunohistochemistry (IHC)

IHC was performed using standard methods and as previously described [16]. For mice treated with lorcaserin (7 mg/kg, IP) or saline (IP) for cFOS and GFP analysis, the perfusion procedure occurred 90 min after treatment. Briefly, under deep terminal pentobarbital anesthesia, mice were transcardially perfused with phosphate buffered saline (PBS) followed by 4% paraformaldehyde (PFA). Brains were extracted, post-fixed in 4% PFA at 4 °C, cryoprotected in 30% sucrose at 4 °C and then sectioned coronally on a freezing sliding microtome at 25 μm (Bright solid state freezer series 8000, Bright Instruments). Tissue was processed for single- or dual-IHC using published methods [16] with the following antibodies: rabbit anti-Pomc primary antibody (1:1,000; H-029-30, Phoenix Pharmaceuticals, Burlingame, CA USA) and an Alexa Fluor 594 donkey anti-rabbit secondary antibody (1:500, A-2127, ThermoFisher Scientific); rabbit anti-*c-fos* primary antibody (1:300, sc-52, Santa Cruz) followed by Alexa Fluor 594 donkey anti-rabbit secondary antibody (1:500, A-2127, ThermoFisher Scientific); and chicken anti-GFP primary antibody (1:800, ab101863, Abcam) followed by Alexa Fluor 488 donkey anti-chicken secondary antibody (1:800, 703-545-155, Jackson ImmunoResearch). Sections were mounted onto microscope slides and coverslipped in an aqueous mounting medium (Vectastain; Vector Laboratories). Slides were imaged on an AXIOSKOP2 (Zeiss) microscope. For single- and dual-IHC expression, the boundaries of the arcuate nucleus of the hypothalamus (ARC) were defined using neuroanatomical landmarks and the Mouse Brain Atlas [28] and analyzed −1.46 to −2.18 from Bregma.

### 2.8 Statistics

Data were analyzed with t-test or One-way, Two-way or Repeated Measures ANOVA followed by Tukey's or Bonferroni *post hoc* tests. For all analyses, significance was assigned at p < 0.05. Data are presented as mean ± SEM.

## 3 Results

### 3.1 Lorcaserin directly improves glucose and insulin tolerance

To investigate whether 5-HT_2C_R agonist and obesity medication lorcaserin has effects on glycemic control that may be dissociated from food intake and body weight effects, WT mice were fed a 60% fat diet for at least 12 weeks to produce a mouse model of T2D. Next, using a within-subjects experimental design, mice were administered a single treatment of saline or lorcaserin (2.5, 5.0 or 10.0 mg/kg, IP) 45 min prior to a glucose tolerance test (GTT; 1 g/kg glucose, IP). Lorcaserin produced a dose-dependent improvement in glucose tolerance, with 5 and 10 mg/kg significantly improving glucose tolerance compared to saline (F_3, 14_ = 11.32, p = 0.005; Figure 1A). Lorcaserin (5.0 and 10.0 mg/kg, IP) also significantly improved insulin sensitivity as measured with an insulin tolerance test (ITT; 0.75 U/kg insulin, IP) in another cohort of DIO WT mice using a within-subjects experimental design (F_3,19_ = 7.08, p = 0.002; Figure 1B). These data indicate that lorcaserin significantly augments glycemic control in a mouse model of T2D.

We next considered the anatomical localization of 5-HT_2C_Rs performing this glucoregulatory function. 5-HT_2C_Rs are expressed in numerous brain regions, including the ARC [29]. To facilitate visualization of 5-HT_2C_Rs within the ARC, a *5-HT*_*2C*_*R*^*CRE*^ line intercrossed with a B6.129X1-*Gt(ROSA)26Sortm1(EYFP)Cos*/J (*Rosa26*^*YFP*^) line was used (Figure 1C). Using IHC, 5-HT_2C_R^CRE:YPF^ expression was mapped within the ARC, which revealed that 5-HT_2C_Rs are expressed throughout the rostral to caudal extent of the ARC (Figure 1D,E). *5-HT*_*2C*_*R*^*CRE:YPF*^ mice treated with lorcaserin (7 mg/kg; Figure 1G) showed a significant increase in a marker for neuronal activity c-Fos immunoreactivity (IR) within ARC GFP-IR cells (15.1 ± 1.7% of GFP-IR cells co-expressed FOS-IR) compared to saline treatment (5.2 ± 1.0% of GFP-IR cells co-expressed FOS-IR; t (11) = 8.41, p < 0.0001; Figure 1F). These data suggest that doses of lorcaserin that improve glycemic control influence the activity of a subset of ARC 5-HT_2C_R-expressing cells. We next evaluated the neurochemical phenotype of ARC 5-HT_2C_R-expressing cells. Consistent with previous reports [16], [30], we observed approximately 40% of POMC-IR neurons express 5-HT_2C_Rs (GFP-IR) (Figure 1H).

Taken together, these data provide evidence for a new glucoregulatory target (5-HT_2C_Rs) that has immediate translational relevance through the use of a medication already in clinical use for obesity treatment, the 5-HT_2C_R agonist lorcaserin.

### 3.2 POMC^ARC:5-HT2CR^ is sufficient to perform lorcaserin's glucoregulatory function

**Figure 2:**
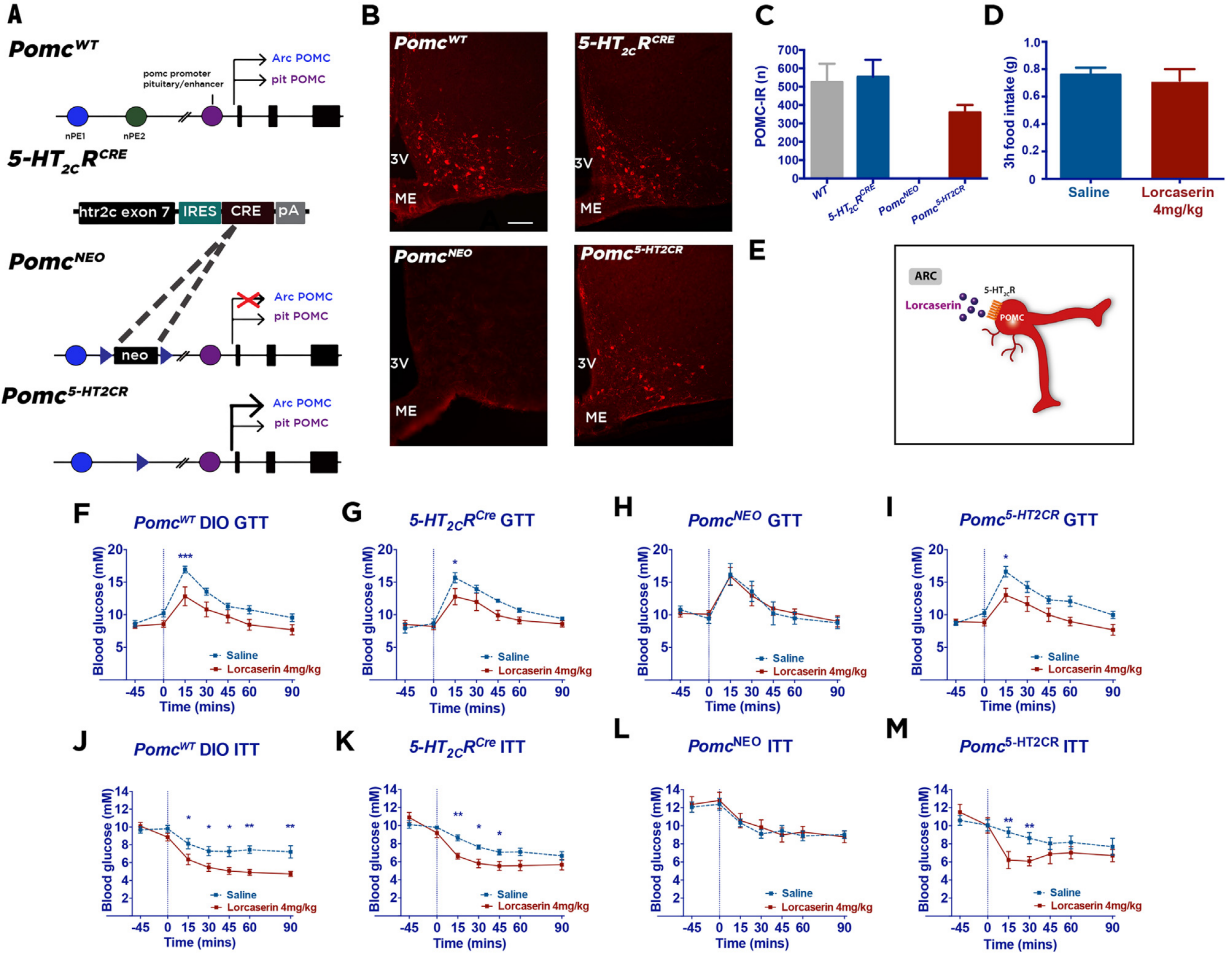
**ARC Pomc**^**5-HT2CR**^**is sufficient to perform lorcaserin's glucoregulatory function.** (A) A reversible ARC *Pomc* knock-out model (*Pomc*^*NEO*^) was intercrossed with a *5-HT*_*2C*_*R*^*CRE*^ line to restore *Pomc* expression specifically within 5-HT_2C_Rs expressing (*Pomc*^*5-HT2CR*^) neurons. (B) Representative images and (C) quantification of average bilateral Pomc-derived peptide immunoreactive (IR) neurons in the ARC (−1.46, −1.58, −1.7, −1.82, −1.94 −2.06 and −2.18 mm from bregma) in male *Pomc*^*WT*^, *5-HT*_*2C*_*R*^*CRE*^, *Pomc*^*NEO*^ and *Pomc*^*5-HT2CR*^ littermates (n = 4 per genotype). (D) Lorcaserin (4 mg/kg, IP) has no effect on food intake compared to saline in male mice (n = 8). (E) Schematic of lorcaserin action at ARC Pomc^5-HT2CR^ neurons. Pretreatment with lorcaserin (4 mg/kg, IP) significantly improves glucose tolerance (GTT; 1 g/kg, IP) and insulin tolerance (ITT; 0.75U/kg, IP) in 6 h light cycle fasted (F, J) DIO male *Pomc*^*WT*^ and (G, K) *5-HT*_*2C*_*R*^*CRE*^ control mice. These effects are abolished in (H,L) male *Pomc^NEO^* mice and restored in (I, M) male *Pomc*^*5-HT2CR*^ mice (n = 7–12 per genotype). Scale bar in B, 100 μm applies to all genotypes. *p < 0.05, **p < 0.01, ***p < 0.001 lorcaserin versus saline treatment.

Given that 5-HT_2C_Rs are anatomically positioned to influence multiple neurochemically defined cell types within the CNS, we next sought to evaluate the specific contribution of POMC^ARC^ peptides to the glucoregulatory effects of lorcaserin. A previous report genetically manipulating 5-HT_2C_R expression indicates that 5-HT_2C_Rs co-expressed with POMC neurons is sufficient to mediate preclinical 5-HT_2C_R agonist's glycemic effects in mice [5]. However, the neurochemical mediator expressed within these neurons has not been established. We specifically investigated whether POMC^ARC^ peptides are a neurochemical communicator of the therapeutic effect of lorcaserin. To selectively manipulate POMC^ARC^, we utilized a reversible ARC *Pomc* knockout model (*Pomc*^*NEO*^) crossed with a *5-HT*_*2C*_*R*^*CRE*^ line to restore *Pomc* expression specifically within 5-HT_2C_Rs expressing (*Pomc*^*5-HT2CR*^) neurons in the ARC (Figure 2A). As expected, *Pomc*^*NEO*^ mice had no detectable hypothalamic POMC-IR, whereas POMC-IR was restored by 40% in *Pomc*^*5-HT2CR*^ mice (F_3, 12_ = 12.91, p = 0.0005; Figure 2B,C). Functionally, genetic inactivation of *Pomc*^ARC^ promotes insulin resistance, but normal glucose levels, primarily by increasing glycosuria in *Pomc*^*NEO*^ mice [31]. The hyperinsulinemia phenotype is normalized by the restoration of *Pomc* in the subset of cells expressing 5-HT_2C_Rs in *Pomc*^*5-HT2CR*^ mice, indicating that *Pomc* synthesized in 5-HT_2C_R cells is sufficient to mediate POMC's effects on insulin sensitivity [16]. We therefore hypothesized that lorcaserin improves insulin sensitivity without altering energy homeostasis or body weight via influencing the release of POMC^ARC^ peptides (Figure 2E), and without functional POMC^ARC^, lorcaserin's glycemic effects would not be achieved.

To address this hypothesis, we employed a dose of lorcaserin (4 mg/kg, IP) that was not sufficient to decrease food intake in DIO WT mice (t (12) = 0.41, p = 0.66; Figure 2D) or body weight (t (13) = 0.12, p = 0.46; Figure S1) and used this dose for glucose and insulin tolerance tests using a within-subjects experimental design. Lorcaserin (4 mg/kg, IP) significantly improved glucose tolerance in both DIO *Pomc*^*WT*^ (treatment F_1, 108_ = 28.61, p = 0.0001; Figure 2F; Figure S2A) and *5-HT*_*2C*_*R*^*CRE*^ control mice (treatment F_1, 87_ = 13.02, p = 0.0005; Figure 2G; Figure S2B); this effect was abolished in POMC deficient *Pomc*^*NEO*^ mice (treatment F_1, 70_ = 0.05, p = 0.82; Figure 2H; Figure S2C) but restored in *Pomc*^*5-HT2CR*^ mice (treatment F_1, 108_ = 27.41, p = 0.0001; Figure 2I; Figure S2D). In a separate cohort of mice, we observed that lorcaserin (4 mg/kg, IP) also significantly improved insulin tolerance in *Pomc*^*WT*^ DIO (treatment F_1, 140_ = 42.04, p = 0.0001; Figure 2J; Figure S2E) and *5-HT*_*2C*_*R*^*CRE*^ control mice (treatment F_1, 126_ = 22.40, p = 0.0001; Figure 2K; Figure S2F); this effect was absent *Pomc*^*NEO*^ mice (treatment F_1, 119_ = 0.31, p = 0.57; Figure 2L; Figure S2G) but restored in *Pomc*^*5-HT2CR*^ mice (treatment F_1, 76_ = 8.52, p = 0.0046; Figure 2M; Figure S2H). Thus, these results identify a specific neurochemical mediator of lorcaserin's glucoregulatory effects, brain POMC^ARC^ peptides. Moreover, these data indicate that POMC in a specific subset of ARC neurons expressing 5-HT_2C_Rs is sufficient to mediate lorcaserin's glycemic effects.

### 3.3 MC4R^ChAT^ is sufficient to mediate lorcaserin's glucoregulatory, but not anorectic, effects

**Figure 3:**
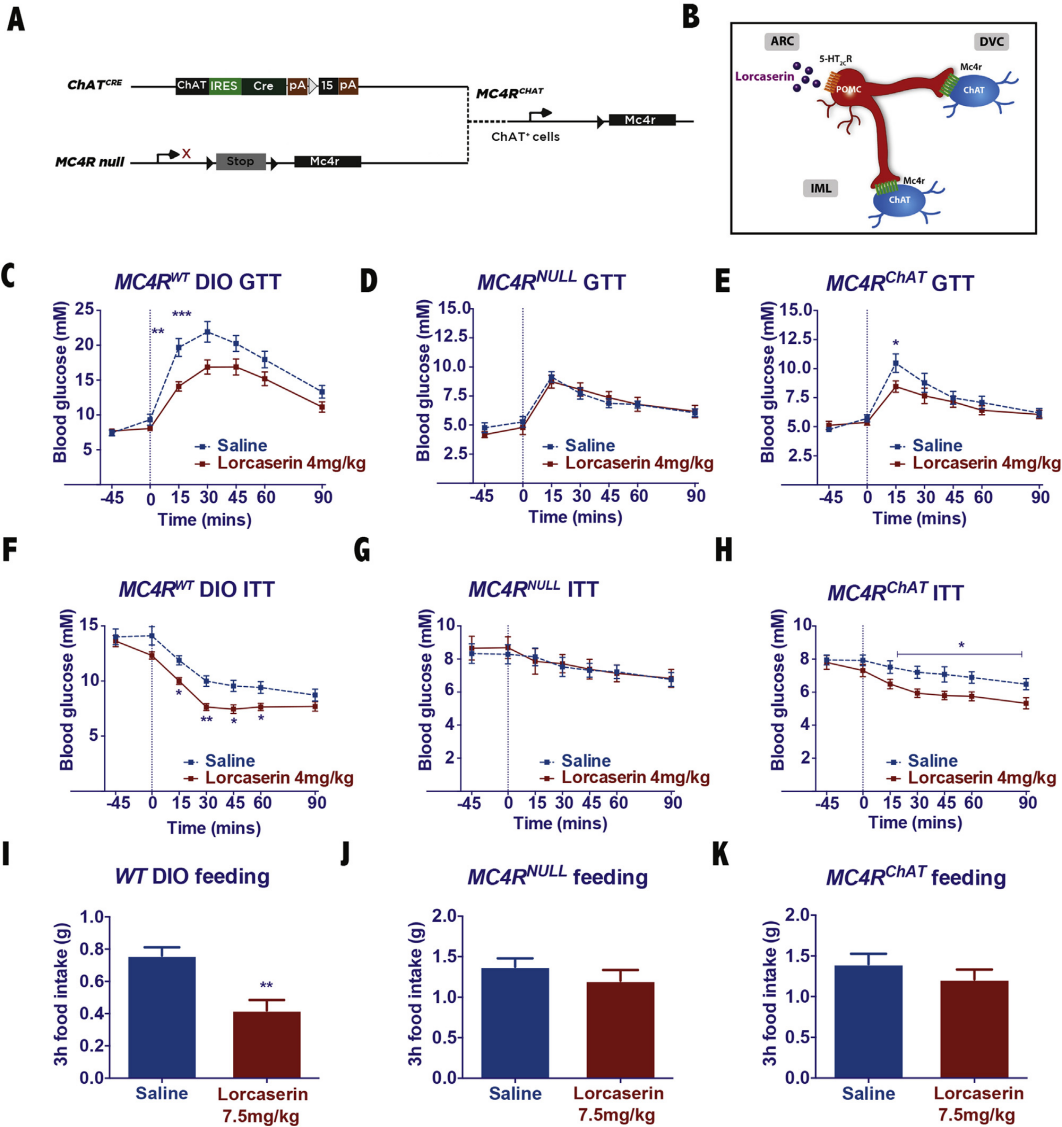
**MC4Rs in cholinergic neurons are sufficient to mediate lorcaserin's glucoregulatory effects but do not communicate lorcaserin's anorectic effects.** (A) The reversible *Mc4r* knockout line (*Mc4r*^*NULL*^) was crossed with a *Choline acetyltransferase* (*ChAT)*^*CRE*^ line to restore *Mc4r* expression specifically within preganglionic sympathetic and parasympathetic neurons (*Mc4r*^*ChAT*^). (B) Schematic of lorcaserin downstream action at cholinergic MC4Rs. (C, F) Lorcaserin pretreatment (4 mg/kg, IP) improves glucose (GTT; 1 g/kg; IP) and insulin tolerance (ITT; 0.75 U/kg, IP) in 6 h light cycle fasted DIO WT male mice. (D,G) These effects are abolished in *Mc4r*^*NULL*^ male mice and (E, H) restored in *Mc4r*^*ChAT*^ male littermates (n = 11–12/genotype). A higher concentration of lorcaserin (7.5 mg/kg, IP) significantly suppresses feeding in (I) WT DIO mice but does not influence feeding in (J) *Mc4r*^*NULL*^ or (K) *Mc4r*^*ChAT*^ male littermates (n = 7–8/genotype). *p < 0.05, **p < 0.01, ***p < 0.001 lorcaserin versus saline treatment.

We next examined the receptor target of POMC^ARC^ peptides communicating lorcaserin's glucoregulatory effect. POMC^ARC^ peptides signal at a family of CNS receptors. Previous reports indicate that brain inactivation of the melanocortin-4 receptor (MC4R) subtype promotes hyperphagia, obesity, and a T2D phenotype, attenuates preclinical 5-HT_2C_R agonist's anorectic and glycemic effects, and that MC4Rs specifically within cholinergic neurons play a physiological role in the regulation of hepatic insulin sensitivity [11], [24], [25]. Specifically, a reversible *Mc4r* knockout mouse (*Mc4r*^*NULL*^) intercrossed with a *Choline acetyltransferase* (*ChAT;* the enzyme that carries out acetylcholine production*)*^*CRE*^ line to restore *Mc4r* expression exclusively within preganglionic sympathetic and parasympathetic neurons (*Mc4r*^*ChAT*^; Figure 3A) revealed that *Mc4r*^*NULL*^ mice are hyperinsulinemic and hyperglycemic and that restoration of the MC4Rs in ChAT cells in *Mc4r*^*ChAT*^ mice improves hyerinsulinemia, hyperglycemia, hepatic insulin action, and insulin-induced suppression of hepatic glucose production, without normalizing hyperphagia [25]. We therefore hypothesized that the melanocortin receptor mediating lorcaserin's glycemic effects via POMC^ARC:5-HT2CR^ activation is the MC4R, and the specific subset of MC4Rs is those modulating autonomic outflow via expression on cholinergic preganglionic neurons (Figure 3B).

To directly assess the role of cholinergic MC4Rs in lorcaserin's glycemic effects, we performed GTTs and ITTs as described above in DIO *Mc4r*^*WT*^, *Mc4r*^*NULL*^, and *Mc4r*^*ChAT*^ mice (Figure 3A). Although lorcaserin (4 mg/kg, IP) improved glycemic control in DIO *Mc4r*^*WT*^ mice (GTT F_1, 149_ = 26.06, p = 0.0001; ITT F_1, 98_ = 36.86, p = 0.001; Figure 3C,F; Figure S3A,D), it was ineffective in altering glucose (F_1, 119_ = 0.07, p = 0.78; Figure 3D; Figure S3B) or insulin (F_1, 84_ = 0.12, p = 0.74; Figure 3G; Figure S3E) tolerance in *Mc4r*^*NULL*^ mice. However, lorcaserin produced a significant improvement in both glucose (treatment F_1, 154_ = 5.11, p = 0.025; Figure 3E) and insulin (treatment F_1, 127_ = 25.92, p = 0.0001; Figure 3H; Figure S3F) tolerance in mice in which MC4Rs were restored in cholinergic neurons (*Mc4r*^*ChAT*^). Note that although lorcaserin significantly improved glucose tolerance when analyzed across time (Figure 3E), when analyzed as area under the curve (Figure S3C), the difference between treatment groups did not reach statistical significance. This may be a result of methodological factors (e.g. sample size) or may be due to a smaller role of MC4R^ChAT^ in lorcaserin's effects on glucose tolerance. These data reveal that CNS MC4Rs are necessary for lorcaserin's glucoregulatory effects and that MC4Rs expressed in cholinergic neurons are sufficient to mediate at least some of these effects.

We also sought to evaluate the contribution of cholinergic MC4Rs to lorcaserin's anorectic effects. As expected, lorcaserin (7.5 mg/kg, IP) reduced food intake in WT DIO mice (t (14) = 4.1, p = 0.001; Figure 3I). Like lorcaserin's glucoregulatory effects, we observed that downstream activation of CNS MC4Rs is required for lorcaserin to decrease feeding (t (11) = 1.03, p = 0.34; Figure 3J). However, restoration of the subset of MC4Rs expressed in cholinergic neurons in *Mc4r*^*ChAT*^ mice was not sufficient to restore lorcaserin's anorectic effects (t (11) = 1.04, p = 0.32; Figure 3K). These results indicate that while lorcaserin requires downstream CNS MC4R activation to promote both glycemic and hypophagic effects, the subpopulation of MC4Rs mediating these effects is different. Lorcaserin engages non-cholinergic MC4Rs to elicit its effects on feeding and cholinergic MC4Rs to improve glucose homeostasis.

### 3.4 Lorcaserin improves insulin sensitivity, suppresses hepatic glucose production, and increases glucose disposal, but does not increase insulin secretion

**Figure 4:**
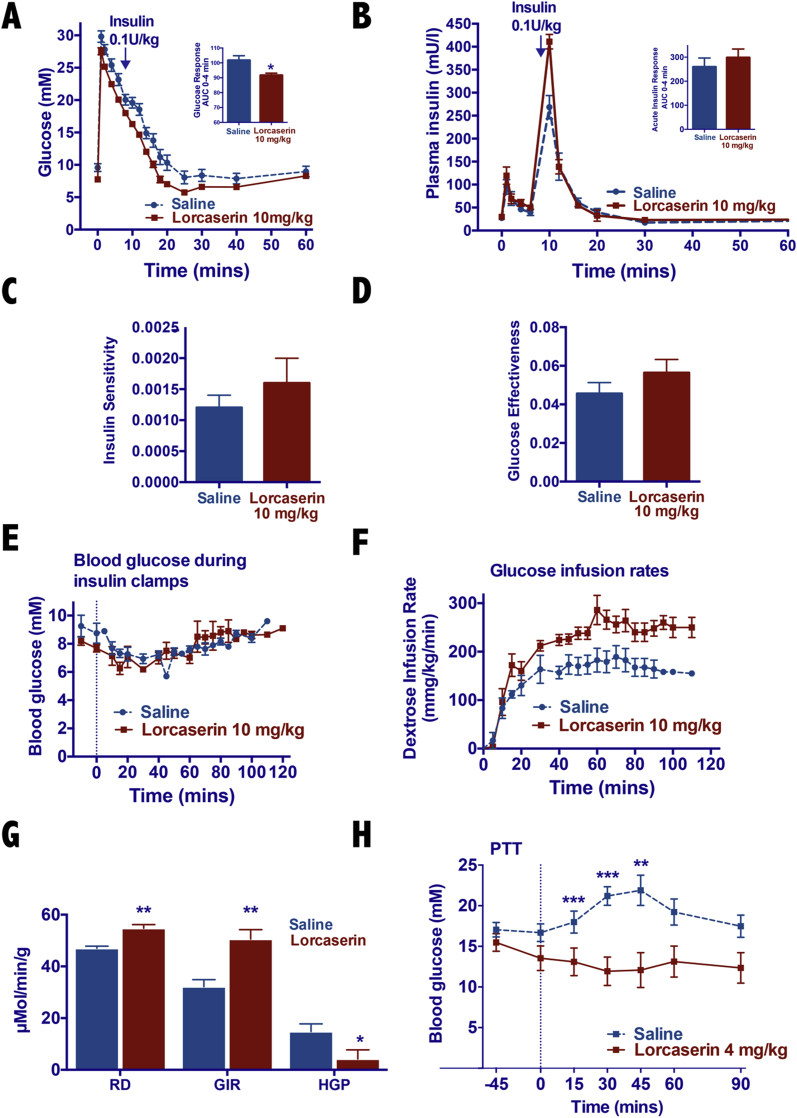
**Lorcaserin suppresses hepatic gluconeogenesis but does not affect insulin secretion in mouse model of DIO T2D.** (A) Blood glucose, (B) plasma insulin, (C) insulin sensitivity, and (D) glucose effectiveness during frequently sampled intravenous glucose tolerance test (FSIVGTT) following pretreatment with lorcaserin (10 mg/kg, IP) in male DIO WT mice. (E) Blood glucose, (F) glucose infusion rates (GIR), (G) average disposal rate (RD), average GIR, and average hepatic glucose production (HGP) during hyperinsulinemic-euglycemic clamps in male DIO WT mice. (H) Pretreatment with lorcaserin (4 mg/kg, IP) improves pyruvate tolerance (PTT; 1.5 g/kg, IP) in 6 h light cycle fasted DIO WT mice (n = 7–10). *p < 0.05, **p < 0.01, ***p < 0.001 lorcaserin versus saline treatment.

Having established a melanocortin neurocircuit that is both necessary and sufficient to mediate the glucoregulatory effects of lorcaserin, we next sought to characterize the downstream peripheral mechanisms underpinning lorcaserin's glycemic effects. Peripheral 5-HT is co-localized with insulin in pancreatic beta cells and promotes insulin's release [32]. We first investigated whether the 5-HT_2C_R agonist lorcaserin may affect insulin secretion by performing intravenous glucose tolerance tests, sampling blood frequently via an indwelling arterial catheter from freely-moving DIO WT mice following delivery of an intravenous glucose bolus. Lorcaserin improved glucose clearance (area under glucose curve) following glucose administration (t (19) = 2.54, p = 0.01; Figure 4A), but we did not detect any effects of lorcaserin on acute insulin response to glucose (t (19) = 0.97, p = 0.34; Figure 4B). The improvement we observe in glucose tolerance in response to lorcaserin cannot be attributed to increased insulin secretion.

Modeling insulin sensitivity from insulin and glucose data collected during the intravenous glucose tolerance tests following a bolus of exogenous insulin delivered 9 min after the glucose bolus, we observed a non-significant trend for improved insulin sensitivity and perhaps glucose effectiveness (Figure 4C,D). This may be in part related to the limitations in sampling frequency and speed and the sampling volumes required during intravenous glucose tolerance tests in mice compared with humans. To examine insulin sensitivity in more detail, we performed insulin clamps with isotope measures of glucose turnover. In keeping with our previous data consistently showing an effect of lorcaserin to improve insulin tolerance, DIO WT mice treated with lorcaserin needed significantly higher rates of dextrose infusion during clamps to maintain blood glucose (treatment F_1, 166_ = 93.2, p = 0.0001; Figure 4E,F). This was explained by both an increase in glucose disposal (t (7) = 4.50, p < 0.01) and a greater suppression of hepatic glucose production (t (8) = 9.02, p < 0.01) under clamp conditions (Figure 4G).

The effects observed on hepatic glucose production are consistent with previous data showing that under physiological conditions, the central melanocortin pathway plays a role in the suppression of endogenous glucose production [25], [33]. To further examine lorcaserin's effects on gluconeogenesis, 6 h fasted DIO WT mice were administered a single injection of lorcaserin (4 mg/kg, IP) or vehicle 45 min prior to a tolerance test using the exclusive hepatic gluconeogenic substrate pyruvate (1.5 g/kg, IP) [34], [35]. Whereas pyruvate administration elicited a marked glycemic excursion in saline pretreated mice, lorcaserin pretreatment completely abolished glucose production upon pyruvate administration (F_1, 98_ = 46.0, p = 0.0001; Figure 4H). Taken together, our data suggest that lorcaserin improves glucose homeostasis acting centrally via POMC^ARC^ signaling to MC4R^ChAT^ to increase autonomic outflow to improve hepatic insulin sensitivity, reduce hepatic glucose production, and improve glucose disposal.

## 4 Discussion

T2D is a chronic disease whose prevalence continues to escalate on a global scale, thereby highlighting the clinical need for a diverse battery of treatment options. Current frontline medications for T2D target peripheral tissues to produce improvements in blood glucose and insulin function [36], [37]. Here we investigate the potential of targeting 5-HT_2C_R circuits within the brain to improve glucoregulatory function and use specific target manipulations to clarify the neurocircuitry underpinning the therapeutic effects.

Lorcaserin is currently licensed for weight loss. In clinical practice in T2D, weight loss is generally associated with improved glycemic control making it difficult to separate lorcaserin's effects on glycemia from its anorectic actions [38], [39], [40]. Using a targeted murine approach, we demonstrate that lorcaserin improves glycemic control via a neurocircuit discrete from that which mediates its effects on feeding or body weight.

To specifically probe how this effect may be achieved, we focused on the homeostatic brain region, the ARC. Within the ARC, POMC neurons are glucose sensing and have been implicated in the control of insulin sensitivity [31], [41], [42]. Specifically, POMC^ARC^ deficient mice exhibit normal blood glucose despite extreme insulin resistance [16], [31] due to elevated glycosuria via reduced sympathetic nerve activity to the kidney and decreased GLUT2 [31]. Restoration of *Pomc* expression only in cells with 5-HT_2C_Rs corrects hyperinsulinemia and insulin sensitivity [16]. In addition, 5-HT and 5-HT_2C_R agonists depolarize POMC^ARC^ neurons [14], [15], [17] and preclinical 5-HT_2C_R agonists improve glycemia by acting at 5-HT_2C_R expressed on POMC neurons [5], [18]. A recent report suggests that this is via action at transient receptor potential cation 5 (TrpC5) [43]. However, the role of POMC peptides synthesized within these neurons in lorcaserin's effects has not been investigated. We found that lorcaserin's glucoregulatory effects were absent in mice lacking hypothalamic *Pomc* and that restoration of *Pomc* in 5-HT_2C_R-expressing neurons was sufficient to rescue lorcaserin's glycemic actions. These data suggest that ARC POMC^5-HT2CR^ is necessary for the glycemic actions of lorcaserin.

Downstream, POMC peptides primarily act via MC4Rs to elicit anorectic and glucoregulatory effects [44], [45], [46], [47]. In agreement with a previous report using preclinical 5-HT_2C_R agonists [30], we observed that lorcaserin's glucoregulatory effects were abolished with global MC4R deficiency in mice. To probe the specific subpopulation of MC4Rs mediating this effect, we employed a genetic approach. Under physiological conditions, forebrain single-minded homolog 1 (SIM-1) MC4Rs have been implicated in feeding behavior [48], whereas cholinergic MC4Rs mediate melanocortin's glycemic effects [25]. Consistent with these divergent physiological roles for MC4R subpopulations, we found that restoration of cholinergic MC4Rs was sufficient to mediate lorcaserin's effects on glucose homeostasis but not feeding. Taken together, these data and our current findings support a divergence between MC4Rs mediating 5-HT obesity compounds' effects on feeding and glycemia.

Within the CNS, the two sites of MC4R and ChAT co-expression are within the preganglionic parasympathetic neurons of the dorsal motor nucleus of the vagus (DMV) and the pre-ganglionic sympathetic neurons of the intermediolateral nucleus (IML) of the spinal cord. Our findings are consistent with earlier reports demonstrating a physiological role for cholinergic MC4Rs within the IML in glucose homeostasis [25], [33]. Furthermore, pre-clinical 5-HT_2C_R agonist administration is associated with an increase in cholinergic neuronal activation within the IML, POMC^ARC^ neurons project to the IML and MC4R agonists depolarize these same neurons [24], [49]. While it cannot be excluded that cholinergic MC4Rs in the DMV contribute to lorcaserin's glucoregulatory effects, physiologically, DMV neurons have been reported to regulate insulin secretion rather than insulin sensitivity, MC4Rs in the DMV are inhibited by MC4R agonists and do not appear to be regulated by 5-HT_2C_R agonists [11], [24], [25], [33]. On balance, therefore, we postulate that MC4Rs in IML cholinergic neurons are a critical component of the neural circuit through which lorcaserin achieves its glycemic effects.

The absence of POMC signaling in *Pomc*^*NULL*^ mice leads to reduced sympathetic nerve activity to the kidney and as a result, a downregulation of GLUT2, and extreme insulin resistance without elevated glucose [31]. The proposed effects of lorcaserin via POMC^ARC^ to IML MC4R^ChAT^ therefore appear at odds with this phenotype. There could be many reasons for this apparent discrepancy, such as differential innervation of IML and DMV cholinergic neurons by 5-HT_2C_R positive and negative POMC^ARC^ neurons and competing actions of parasympathetic and sympathetic autonomic outflow to multiple tissues on glucose regulation. Alternatively, the difference in results may be due simply to a difference in physiological effect via a loss (*Pomc*^*NULL*^) or gain (activation through lorcaserin) of function. Further delineation of the specific subset of MC4R^ChAT^ neurons underpinning lorcaserin's glycemic effects requires additional investigation.

Our insulin clamp data suggest that the peripheral mechanisms through which lorcaserin acting via brain autonomic outflow elicits its anti-diabetic effects include both suppression of hepatic glucose production and increased glucose uptake but that lorcaserin does not appear to act by altering insulin secretion. The former is consistent with earlier reports emphasizing the role of the melanocortin circuit in the control of peripheral blood glucose via the regulation of hepatic glucose production, with mice lacking 5-HT_2C_Rs displaying hepatic insulin resistance and resistance to the anti-diabetic effects of 5-HT_2C_R compounds, both of which were restored when 5-HT_2C_Rs were re-expressed in POMC neurons [5]. MC4Rs specifically in cholinergic neurons have also been demonstrated to regulate the ability of insulin to suppress hepatic glucose production [25].

## 5 Conclusions

In conclusion, T2D is a chronic, debilitating disease with major social, fiscal, and medical costs. The prevalence of this disease is increasing markedly, and, despite the emergence of new therapies targeting insulin secretion, insulin action and/or glucose excretion, glycemic control remains generally sub-optimal, emphasizing the continued need for new therapeutic options. Given the data supporting a key role for brain in control of glucose homeostasis [2], [3], [4], [5], [6], [7], [8], [9], [50] and the reports of lorcaserin improving glycemic control in patients with T2D with weight loss, we utilized a molecular and genetic approach to describe a POMC^ARC:5-HT2CR^ to MC4R^ChAT^ glucoregulatory neurocircuit. Moreover, we report the clinical relevance of this by demonstrating that lorcaserin acting at the level of the hypothalamus on this POMC^ARC:5-HT2CR^ to MC4R^ChAT^ pathway improves peripheral glucose homeostasis in a mouse model of T2D. There is growing interest in brain control of peripheral glycemia and the potential for targeting the ARC for therapy in T2D [51]. Our work suggests that lorcaserin should be investigated in patients with T2D for blood glucose lowering therapy, in addition to its already approved licensing for body weight reduction.

## Author contributions

LKB, MLE, and LKH conceived the study and designed experiments. LKB, CC, PBMM, LVT, TG, and GD'A performed experiments in Figure 1. LKB performed experiments displayed in Figure 2, Figure 3, Figure 4 with help from EO-B, TG, NH, YR, CR and GD'A. MR, MJL, MGM, and JJR provided reagents and shared expertise. LKB, MLE, and LKH wrote the manuscript with input from all other authors.
