# Supplementary material for: Lorcaserin improves glycemic control via a melanocortin neurocircuit
Source: Mol Metab. 2017 Jul 21;6(10):1092–102. doi: 10.1016/j.molmet.2017.07.004 (PMC5641625; doi:10.1016/j.molmet.2017.07.004)
Supplement: Supplementary file 1 [file mmc1.docx]

**Lorcaserin improves glycemic control via a melanocortin neurocircuit**

Luke K. Burke^1,2^†, Emmanuel Ogunnowo-Bada^2^, Teodora Georgescu^3^, Claudia Cristiano^3^,

Pablo B. Martinez de Morentin^3^, Lourdes Valencia Torres^1,3^, Giuseppe D’Agostino^1,3^,

Christine Riches^2^, Nicholas Heeley^2^, Yue Ruan^2^, Marcelo Rubinstein^4^, Malcolm J. Low^5^,

Martin G. Myers^6^, Justin J. Rochford^3^, Mark L. Evans^2*^, Lora K. Heisler^1,3*^

**Supplemental Figures**

**Figure S1. Identification of dose of lorcaserin that does not impact food intake or body weight.** Lorcaserin (4 mg/kg, IP) does not impact 24 hour (A) food intake or (B) body weight compared to saline treatment in male mice (n=7-8/treatment).

**Figure S2. POMC^ARC:5-HT2CR^** **is sufficient to perform lorcaserin’s glucoregulatory function.** Pretreatment with lorcaserin (4 mg/kg, IP) significantly improves area under the curve (AUC) glucose tolerance (GTT; 1 g/kg, IP) and insulin tolerance (ITT; 0.75U/kg, IP) in 6 hour fasted (A,E) DIO *Pomc^WT^* and (B,F) *5HT_2C_R^Cre^* control mice. These effects are abolished in (C,G) Pomc^NEO^ mice and restored in (D,H) Pomc^5HT2CR^ mice (n=7-12 per genotype). * p < 0.05, ** p < 0.01 lorcaserin versus saline treatment.

**Figure S3. MC4Rs in cholinergic neurons are sufficient to mediate lorcaserin's glucoregulatory effects.** (A,D) Lorcaserin pretreatment (4 mg/kg, IP) improves glucose (GTT; 1 g/kg, IP; 12 hour dark cycle fast) and insulin tolerance (ITT; 0.75 U/kg, IP; 6 hour light cycle fast) as measured by area under the curve (AUC) in DIO *Mc4r^WT^* mice. (B,E) These effects are abolished in *Mc4r^NULL^* mice and (E,H) restored in ITT of *Mc4r^ChAT^* littermates (n=11-12/genotype). ** p < 0.01 lorcaserin versus saline treatment.
